# The past, present and future of Alzheimer's disease – part 1: the past

**DOI:** 10.1055/s-0043-1777722

**Published:** 2023-12-29

**Authors:** Ricardo Nitrini

**Affiliations:** 1 Universidade de São Paulo, Faculdade de Medicina, São Paulo SP, Brazil.

**Keywords:** History, Alzheimer Disease, Plaque Amyloid, Neurofibrillary Tangles, Amyloid beta-Peptides, Alois Alzheimer, Oskar Fischer, Emil Kraepelin, História, Doença de Alzheimer, Placa Amiloide, Emaranhados Neurofibrilares, Peptídeos beta-Amiloides, Alois Alzheimer, Oskar Fischer, Emil Kraepelin

## Abstract

**Background** Alzheimer's disease (AD) was described in 1907, and since then it changed from a relatively rare condition to one of the most prevalent diseases.

**Objective** To describe the evolution of the notions of dementias and AD, and to investigate the reasons for the increase in scientific interest in AD.

**Methods** A historical analysis was carried out on knowledge about dementia, the site of mental activity, the relationships between brain diseases and mental activity, and on the advances in research about AD, since its discovery until the publication of the amyloid cascade hypothesis in 1992. A search was carried out in the National Library of Medicine (PubMed) for scientific articles that included the terms dementia or AD over 50 years, from 1972 to 2021.

**Results** The scientific research on AD increased from 615 papers with the term AD in the first decade (1972-1981), to 100,028 papers in the last decade (2012-2021): an increase of 162.6 times whereas publications with the term dementia increased 28.6 times in the same period. In the 1960s and 1970s, a consensus was reached that AD is responsible for the majority of cases of dementia previously known as senile dementia. In the 1980s, beta-amyloid peptide was identified in the core of the senile plaque, hyperphosphorylated tau protein was found in neurofibrillary tangles, and a mutation was discovered in a hereditary form of AD.

**Conclusion** The expansion of the concept of AD to include senile dementia, and the discoveries that occurred in the 1980s greatly expanded research in AD.

## Resumo

**Antecedentes** A doença de Alzheimer (DA) foi descrita em 1907 e, desde então, deixou de ser relativamente rara para se tornar uma das doenças mais prevalentes.

**Objetivo** Descrever a evolução das noções sobre demências e DA e investigar as razões do aumento do interesse científico pela DA.

**Métodos** Foi realizada uma análise histórica dos conhecimentos sobre demência, o local da atividade mental, as relações entre doenças cerebrais e a atividade mental, e sobre os avanços na pesquisa sobre a DA, desde a sua descoberta até a publicação da hipótese da cascata amiloide em 1992. Foi realizada uma busca na Biblioteca Nacional de Medicina dos Estados Unidos da América (PubMed) por artigos científicos que incluíssem os termos demência ou DA nos 50 anos, de 1972 a 2021.

**Resultados** A pesquisa científica sobre DA aumentou de 615 artigos com o termo doença de Alzheimer na primeira década (1972-1981), para 100.028 artigos na última década (2012-2021): um aumento de 162,6 vezes enquanto as publicações com o termo demência aumentaram 28,6 vezes no mesmo período. Nas décadas de 1960 e 1970, chegou-se a um consenso de que a DA é responsável pela maioria dos casos de demência, anteriormente conhecida como demência senil. Na década de 1980, o peptídeo beta-amiloide foi identificado no núcleo da placa senil, a proteína tau hiperfosforilada foi encontrada em emaranhados neurofibrilares e uma mutação foi descoberta em uma forma hereditária de DA.

**Conclusão** A expansão do conceito de DA para incluir a demência senil e as descobertas ocorridas na década de 1980 ampliaram enormemente a pesquisa em DA.

## INTRODUCTION

This article is based on a conference given in brain, behavior, and emotions, a multidisciplinary congress held in Florianópolis, Brazil, in June 2023. The multidisciplinary characteristics of the congress made it necessary to give explanations that were abbreviated for this publication.

The title of the article is rather pretentious, but it is also an opportunity for a general and personal view of Alzheimer's disease (AD) and dementias.

The objective is to give a view from the remote past to Alois Alzheimer's discovery, and from that to the period of the 1970s and 1980s when I was in the residency and post-graduate training, learning cognitive neurology and dementia. Then, in part 2, it goes to the present time with its hypotheses and theories, and from these to the near future and, even more cautiously, to the more distant future.

## THE VERY FAR PAST

The decline in mental activity with aging already worried the ancient Egyptians. The Egyptian Vizier Ptahhotep wrote the following verses 2400 years B.C.: “The mouth, silent, does not speak,/ The empty heart, does not remember the past.../ What age does to the people is bad in all respects” (cited by Karenberg and Förstl, 2006). 1

In Rome, Marcus Tulio Cicero (106- 43 B.C.) wrote in De Senectude, in 44 BC: “But, is alleged, the memory is impaired. Of course, if you do not exercise it...”. 2 He therefore recognized that memory decline was not part of normal aging. His view was closer to that currently defended.

## THE SEAT OF MENTAL ACTIVITY

Recognizing the seat of mental activity has always been a complex issue for human beings. This concern was already present among the Egyptians who placed it on the heart and lungs. The heart was recognized as the seat of mental activity by the Babylonians, Indians, and even North American indigenous people. 3 This conception, apparently meaningless today, is based on two simple observations: the first is that life ceases with the heart stopping and the second is that emotions cause sensations in the chest that result from tachycardia. It is recognized that in the fifth century BC, Alcmaeon of Croton in Sicily, which was then part of Magna Grecia, was the first to write that mental activity originated in the brain. Alcmaeon, who is considered a member of the Pythagorean school, carried out dissections and found that the optic nerves went toward the brain (they were light-bearing paths to the brain). Hippocrates and the Hippocratic doctors, contemporaries of Alcmaeon, also located mental activity in the brain. 3 However, Aristotle, approximately a century later, maintained the conception that the heart was primarily responsible for mental activity in its broadest sense. 3 Aristotle exerted a great influence on Western civilization, in such a way that (the heart is still the seat of passions and mind in Western music and poetry).

## THE BRAIN AND MENTAL ILLNESS

Hippocrates and his school already considered mental illnesses and epilepsy as diseases of the brain, 3 but for a long time, scholars of mental illnesses did not directly relate them to the brain. Or the brain was not taken into consideration.

The most important demonstration that mental illnesses were a consequence of brain impairment occurred in 1822, when Antoine-Laurent-Jessé Bayle (1799-1858), then 23 years old, carried out his thesis called *Recherches sur les maladies mentales* in which he studied the General Paresis of the Insane (GPI or Dementia Paralytica) and wrote: “I will achieve my goal if this part of my work can prove that chronic arachnitis exists and that it gives rise to the symptoms of mental derangement”. 4 And he demonstrated the inflammation that affected the meninges and brain parenchyma. “For the first time, then, 'something' had been discovered in the brain of the insane.” 4

The proof that a mental illness as complex as GPI is caused by impairment of the brain received the following comment from Emil Kaeprelin, considered the father of Modern psychiatry, in 1917: “The great step toward understanding the etiology of mental illness was the discovery that paresis (GPI) resulted from syphilis”. 5 It took more than 90 years for demonstrating that GPI is caused by syphilis, when, after the discovery of Treponema pallidum as the causal agent of syphilis by Shaudin and Hoffman in 1905, Noguchi and Moore identified it in the brains of patients with GPI in 1913. 6

Although science has only demonstrated that mental illness can occur due to brain damage, popular culture, and art had already anticipated it, as demonstrated by the painting by Hieronymus Bosch (1450-1516), called The Extraction of the Stone of Madness or The Cure of Folly.

From Bayle's discovery, it became clear that every psychiatric hospital should have a laboratory for histopathological studies of the brain.

## THE DISCOVERY OF ALOIS ALZHEIMER

Alois Alzheimer (1864-1915) was a physician at the Municipal Asylum for the Insane and Epileptic in Frankfurt am Main, when in 1901 he examined for the first time the patient Auguste Deter, a 51-year-old woman with a progressive mental illness that had started about six years before, with excessive jealousy of her husband followed by memory impairment, topographical disorientation, difficulties in carrying out domestic tasks, and delusions that people wanted to kill her when she started screaming or hiding 7 8 When A. Alzheimer first evaluated her In November 1901, Auguste D. already had a moderate to severe dementia syndrome. When examining the patient, A. Alzheimer performed a physical examination and a neurological examination in which he assessed the pupils, reflexes, temporal and spatial orientation, memory, and the presence of aphasia, agnosia, apraxia, and acalculia. There were important mental and cognitive disorders, she seemed to not understand where she was, and she had language and perception disorders in which she did not recognize the use of some objects. There were no motor or gait changes. 7 8 A. Alzheimer was intrigued by the case and in the provisional diagnosis he noted arteriosclerotic brain atrophy with a question mark. 7

Usually, Alois Alzheimer is referred to as a psychiatrist. The evaluation of patient Auguste Deter is more akin to the examination carried out by a physician trained as a neurologist than as a psychiatrist. An explanation is in order here. There were no differences in the training of psychiatrists and neurologists in Germany and German-speaking countries in the 19th century when A. Alzheimer obtained his training and even throughout much of the 20th century. Therefore, A. Alzheimer as well as his contemporaries with the same training should be recognized as neuropsychiatrists. Wilhelm Erb (1840-1921), Kraepelin's mentor, began his fight for the separation of the two specialties in 1880 as he considered it very difficult, if not impossible, for an individual to have sufficient knowledge in both specialties. It is interesting to add that when Emil Kraepelin was invited to take over the chair of Neuropsychiatry at the Berlin Polyclinic, he accepted the invitation as long as another chair was created, that of Neurology. As his request was denied, Kraepelin declined the invitation. 9 10 The separation between the two specialties only occurred in 1970 in Germany. 9

But in addition to being a neuropsychiatrist, A. Alzheimer was an extraordinary neuropathologist. He was known by his contemporaries as “the psychiatrist with the microscope”. 7

His neuropathological studies on GPI received the following commentary in the classic book Neurosyphilis, from 1946: “The next advance in our knowledge of paretic neurosyphilis is to be found in the monumental works of Alzheimer and Nissl in 1904”. 11

It is important here to consider the influence of Franz Nissl (1860-1919) on the formation of A. Alzheimer. 7 12 13 14 Nissl discovered a new staining method, which identifies neurons and is still widely used. 14 They worked together at Municipal Asylum in Frankfurt for 7 years and became friends. When A. Alzheimer got married, Franz Nissl was his best man. 7 13 In addition to having taught how to use his staining method and how to use the camera lucida to draw microscopic images, Nissl has probably taught many other aspects of neuropathology and academic matters, because A. Alzheimer considered Nissl his main mentor. 7 13 In 1903, A. Alzheimer joined Franz Nissl again, who was working under the leadership of Emil Kraepelin at the University Hospital of Heidelberg. At the end of the same year, Kraepelin was invited to be the director of the Royal Psychiatric Clinic in Munich and needed someone to create and direct the institution's Neuropathology laboratory. A. Alzheimer was chosen (while Nissl took over Kraepelin's position in Heidelberg). 7 14 After more than one year of hard work by Kraepelin and Alzheimer, the clinic with its laboratory was opened in 1904. 7 This laboratory would later influence the training of many neuropathologists around the world, such as Lewy, Jakob, Creutzfeldt 7 and Von Economo 15 and in addition to Perusini who published the first cases of Alzheimer's disease with A. Alzheimer (cited by Pepeu, 1987). 16

When A. Alzheimer transferred to Breslau where he assumed the position of Full Professor, he was replaced by Walther Spielmeyer as director of the pathology laboratory, which in the period between the First and Second World Wars was the main neuropathology laboratory, responsible for training the most illustrious neuropathologists around the world. 17

The thesis for qualification or permission to teach in the field of neuropsychiatry, presented in 1904 at the University of Munich, is entitled “The Differential Diagnosis of Progressive Paralysis”. 7

And as head of the laboratory, he received Auguste Deter's brain from Emil Sioli, director of the Municipal Asylum in Frankfurt, when she died in 1906. 7

In the Annual Assembly of the German Association of Psychiatry held in Munich, in 1906, Alfred F. Hoche, an important member of the association, criticized the studies that had sought to interpret mental illnesses based on neuropathology and said that “until now, this hope has been the assumption below all expectations”. 7 A. Alzheimer's response was that his patient Auguste D., from Frankfurt, had died less than 2 weeks ago, after 4 and a half years of illness, and that he had the clinical data, had received the brain and that he intended to document that the neuropathological changes in this case were the cause of his mental disorders. 7 It is not clear whether A. Alzheimer had already been able to stain and examine the slides of Auguste D. in a relatively short time or whether his intuition had made him give this information. As we all know, intuition is a very important part of scientific discoveries.

The description of the clinical case and the neuropathological changes were presented for the first time at the Meeting of the South-West Germany Psychiatrists held in Tübingen, on November 6, 1906. 7 8 In addition to citing the clinical characteristics of the patient August Deter, A. Alzheimer presented slides demonstrating senile plaques and neurofibrillary tangles changes observed with the Bielschowski technique (a silver impregnation technique, modified from the Golgi method). He reported the great cerebral atrophy on macroscopic examination and the neurofibrillary tangles and that as the neurofibrils started to be stained differently from normal neurofibrils, a chemical transformation of the neurofibril substance must have occurred; and that they accumulated a pathological product of metabolism inside the neuron. He further described that scattered throughout the cerebral cortex there were a large number of miliary foci that represent “the site of deposition of a peculiar substance in the cerebral cortex.” 8

The conclusions deserve to be repeated today: “It is evident that we are dealing with a peculiar, little-known disease process.” And continued: “We must not be satisfied to force it into the existing group of well-known disease patterns. It is clear that there exist many more mental diseases than our textbooks indicate”. 8

His presentation was not very successful. There were no comments from the audience or even from the chairman. According to Maurer and Maurer, this caused disappointment in A. Alzheimer, and this may have occurred because at that time the psychiatrists were deeply interested in the new psychoanalytic theory, and Carl Gustav Jung a close collaborator of Sigmund Freud was present at the conference. ^7^ The paper on Auguste Deter case was finally published in 1907. 8

In the 1910 edition of his book entitled Psychiatry: A textbook for students and doctors, Emil Kaepelin uses the name Alzheimer's disease for the first time in the chapter Senile and Pre-senile Dementias. 18 And the paragraph in which it mentions it already suggests that the distinction with senile dementia was not well established. Repeating here, from the English version: “The clinical interpretation of this Alzheimer's disease is still confused.” And Kraepelin went on to write that it may be “a particularly serious form of senile dementia” ... or “a more or less age-independent unique disease process.” 18

## THE RELATIVELY RECENT PAST

In 1972 when I started my residency in Neurology in the Neurology Division of Hospital das Clínicas of the University of São Paulo, Brazil, and, as usual, only a few residents were more interested in what were then called Higher Nervous Activities or Neuropsychology. I was one of these. At that time, we strived for understanding aphasia, memory, agnosias, and apraxias. Dementia was not a very important part of our studies. Aleksander R. Luria 19 20 and Norman Geschwind 21 22 were our main guides. 23

At that time, Alzheimer's disease (AD), as well as Pick's disease were considered rare forms of presenile dementia, and the general public as well as most physicians considered that dementia of the aged was caused by arteriosclerosis. Today it seems a gross error, but it is necessary to remember that, even today, vascular changes are very common in cases diagnosed as AD, and that pure AD is less common than AD associated with other diseases, especially in the very old, as we will see in part 2. Although larger infarcts are not usual in the dementia of the aged, small vessel disease is common, a fact already reported by Kraepelin in 1910. 18

It is interesting to see, in table, the number of published papers that included the terms Alzheimer's disease or dementia for 50 years, in periods of five years, from 1972 until 2021, that were registered in the National Library of Medicine and obtained through the PubMed on October 5, 2023.

It is possible to observe how the research on dementia and especially on AD increased in these 50 years ( Table 1 ). Even when we take into account the increasing number of journals and papers in general over the years, the increase in scientific research on AD and dementia is still amazing. In the first 10 years (1972-1981), 615 papers included the term Alzheimer's disease, increasing to 9,246 in the following decade (1982-2001). This was an increase of 162.6 times whereas publications with the term dementia increased 28.6 times in the same period.

**Table 1:** Papers on Alzheimer's disease and on dementia in general, published by registered periodicals of the National Library of Medicine, Bethesda, Maryland, USA

Years (period of 5 years)	Alzheimer's disease	Dementia
1972-1976	131	1,681
1977-1981	484	2,642
1982-1986	2,424	5,364
1986-1991	6,822	11,865
1992-1996	9,795	15,016
1997-2001	14,876	20,720
2002-2006	20,698	27,664
2007-2011	28,254	36,097
2012-2016	41,403	50,537
2017-2021	58,625	73,315

It is also interesting to note that in the first version (1985) of one of the best books dedicated to Neuropsychology or Higher Cortical Functions, designations that were appropriately replaced by Behavioral and Cognitive Neurology by Marsel Mesulam, 24 there was not a chapter dedicated to dementia or Alzheimer's disease. However, In the second edition, in 2000, there is an 83-page chapter on aging, Alzheimer's disease, and dementia. 25 Many of the neurologists who were previously dedicated to the study of neuropsychology migrated to the field of dementia, bringing with them the clinical and anatomical knowledge that has been so important to recognize or better redefine forms of degenerative dementia, such as Primary Progressive Aphasia, 26 Behavioral Variant of Frontal Dementia, 27 28 or new signs for the clinical diagnosis of these conditions. 29 30

## ARE AD AND SENILE DEMENTIA ONLY ONE OR TWO DIFFERENT DISEASES?

This question was very difficult to answer. During several years, starting in 1907, the year when the Alzheimer's paper about August Detter was published, there had been doubts about the distinction between AD and senile dementia, as Kraepelin had already noticed in 1910. 18

Many neurologists understood that there was also senile dementia, which was a degenerative dementia, but this senile dementia was considered clinically and pathologically different from AD. The most important difference was the presence of focal signs in AD, such as aphasia, agnosia, and apraxia in AD while in senile dementia memory and behavioral symptoms predominated. Of course, these clinical differences should be related to the topography of the pathological changes or to different pathological changes. 31

However, many authors have published papers arguing the pros and cons of the unitary hypothesis. Many of these papers were based on small numbers of cases. After the Second World War, a series with more than 140 cases was published, which showed that there was no reason to separate AD from senile dementia. 32 33

The paper by Roth, Tomlinson, and Blessed (1966) was very important because they used clinical and pathological data to confirm the correlation between the number of senile plaques and dementia severity. 34

Two other papers were also very important. Vladimir Hachinski et al. stated that “The use of the concept calcification of the arteries of the brain to describe mental decline in the aged is probably the most common misdiagnosis in medicine.” 35 And the paper by Robert Katzman explained that AD was not a rare presenile dementia, but it was a common and malignant disease: “a major killer.” 36

Finally, a consensus was reached and the diagnosis of “senile dementia of the Alzheimer type” was used for a few years, and then replaced by early-onset AD and late-onset AD.

The expansion of the concept of AD to include senile dementia together with the progressive aging of the population made AD leave the backstage to become one of the most frequent diseases of our time.

## OSKAR FISCHER AND A CRITICAL COMMENT

Considering that the pathological changes described by A. Alzheimer are now accepted as the cause of both early-onset and late-onset AD (previously known as senile dementia) it is relevant to discuss a paper by Oskar Fischer (1876-1942). In 1907, the same year that A. Alzheimer published his paper describing Auguste Deter's case, Oskar Fischer, who worked in the German School of Prague, 37 38 published an impressive paper where he described the neuropathological changes he had found in cases of senile dementia. 39 Using several staining methods, which included the Bielschowski stain, Oskar Fischer described the claviform proliferation of neurofibrils and senile plaques (which he firstly called “miliary necrosis”, a name that Fischer informed had been given by Redlich when studying the brain of two cases of senile dementia); at the end of the paper, Fischer preferred to name glandular necrosis than miliary necrosis. After finding these changes in one case of senile dementia (which was then also called presbyophrenia) he studied the brains of 16 cases of senile dementia and found abundant miliary necrosis in 12 cases with severe dementia symptoms, while miliary necrosis was not present in 4 cases with mild symptoms. After that, he studied 65 brains (45 with GPI; 10 with various non-organic psychosis, and 10 with normal brains) and did not find these changes. He also studied 7 cases of cerebral atrophy due to lacunar softening and in only one case there was an isolated focus of miliary necrosis that was identical to those described in senile dementia. His conclusions were:

- “In senile dementia there occur very peculiar claviform proliferations in the neurofibrils, which, in this form, have so far been unknown in the brain”;
- “In 'glandular necrosis' we find the most important anatomical substrate of presbyophrenia”.

Would it be reasonable to include Fischer in the eponym of AD? Alzheimer-Fischer's disease or even Fischer-Alzheimer's disease? History and eponyms are not as fair as we would like them to be. 37 38 However, for these two men, the due recognition of their contribution would be very important. While A. Alzheimer was indicated to be a Full Professor in Breslau, then one important Prussian city (which he certainly deserved for all his contributions to neuropsychiatry and neuropathology), Oskar Fischer died in a concentration camp in 1942. 37

## IMPORTANT DISCOVERIES

At the beginning of the 1980s, we believed that everything would be resolved if we knew what is the dense substance that was contained in the core of the senile plaque. And, also, what are the constituents of the neurofibrillary tangles?

In a few years in the 1980's several important discoveries happened. From 1983 several authors participated in a global effort to identify the constituents of the dense structure of the plaques. Here I will cite only a few of them. A protein of the senile plaque core was isolated and its amino acid composition was identified (Allsop et al., 1983). 40 An amyloid protein was identified in the congophilic angiopathy of AD and named βamyloid (Glenner & Wong, 1984). 41 This protein was then identified in the congophilic angiopathy in Down syndrome, which caused the almost profectally phrase: “Assuming the beta protein is a human gene product, it also suggests that the genetic defect in Alzheimer's disease is localized on chromosome 21”(Glenner & Wong, 1984). 42 The presence of βamyloid peptide in plaques of AD and Down syndrome was confirmed (Masters et al., 1985). 43 Three years later, the amyloid precursor protein (APP) was identified on chromosome 21 (St George-Hyslop et al., 1987) 44 One year earlier, tau protein had been identified in neurofibrillary tangles in AD (Grundke-Iqbal et al., 1986) 45 and in the same year it was recognized that tau protein was abnormally phosphorylated in AD (Grundke-Iqbal et al., 1986). 46

Since the 1970's there has been an almost continuous discussion about which was the most important cause of the symptoms of AD: the neuritic plaque or the neurofibrillary tangle. After these discoveries these researchers were classified as Tauists or βaptists according to the side they support. Genetics was a strong support for the βaptists whereas neuropathology, in which there was a sound clinic-pathological relationship with neurofibrillary tangles, gave strength to the tauists. I had the opportunity to read and also to attend vibrant discussions between the two groups in congresses. Among important leaders of these groups, there were Robert D. Terry (1924-2017) 47 a neuropathologist for the tauists, and Dennis J. Selkoe (1943-) 48 a neurologist with a deep interest in biochemistry for the βaptists. At that time it was difficult to adopt a neutral position. 49

In 1992, Hardy and Higgins proposed the Amyloid Cascade Hypothesis that since then has been the strongest theory for the cause of AD. 50 According to this theory, AD is mainly caused by the toxic actions and deposition of the beta-amyloid peptide with 42 amino acids, which is predominantly formed and/or not properly eliminated from the brain in AD. The hyperphosphorylation of tau proteins and the formation of NFT are considered downstream phenomena caused by the depositions of the beta-amyloid peptide, through still unknown pathophysiology,

This brings us to the present times with its theories, and views for the future, to be presented in part 2.
