# Erratum

**DOI:** 10.1055/s-0044-1779607

**Published:** 2024-02-29

In the article “The past, present and future of Alzheimer's disease - part 1: the past,” with DOI code number https://doi.org/10.1055/s-0043-1777722 , published in the Arq Neuropsiquiatr. 2023 Dec;81(12):1070–1076:

In the Table 1 , in row number 4

**Table:** 

Years (period of 5 years)	Alzheimer's disease	Dementia
1972–1976	131	1,681
1977–1981	484	2,642
1982–1986	2,424	5,364
1986–1991	6,822	11,865
1992–1996	9,795	15,016
1997–2001	14,876	20,720
2002–2006	20,698	27,664
2007–2011	28,254	36,097
2012–2016	41,403	50,537
2017–2021	58,625	73,315

It should be:

**Table:** 

Years (period of 5 years)	Alzheimer's disease	Dementia
1972–1976	131	1,681
1977–1981	484	2,642
1982–1986	2,424	5,364
1987–1991	6,005	10,348
1992–1996	9,795	15,016
1997–2001	14,876	20,720
2002–2006	20,698	27,664
2007–2011	28,254	36,097
2012–2016	41,403	50,537
2017–2021	58,625	73,315

